# Bowel preparation for colonoscopy in patients with surgically altered anatomy: a narrative review and institutional protocol

**DOI:** 10.1016/j.igie.2026.05.001

**Published:** 2026-05-19

**Authors:** Fouad Jaber, Mark Peicher, Juan D. Gomez Cifuentes, Maria P. Forero Rios, Robert J. Sealock

**Affiliations:** 1 Division of Gastroenterology and Hepatology, Baylor College of Medicine, Houston, Texas, USA; 2 Internal Medicine Department, University of New Mexico, Albuquerque, New Mexico, USA; 3 Division of Gastroenterology and Hepatology, Presbyterian Healthcare Services, Albuquerque, New Mexico, USA; 4 Department of Medical Education, Universidad El Bosque School of Medicine, Bogotá, Colombia

## Abstract

Colonoscopy is a vital diagnostic and therapeutic procedure for diseases affecting the rectum, colon, and terminal ileum. In patients who have undergone colorectal surgery, postoperative colonoscopy is frequently required for purposes such as colorectal neoplasia surveillance. However, administering bowel preparation in this population poses significant challenges for patients, caregivers, and the endoscopy team, including gastroenterologists and nurses. Despite these challenges, there is limited peer-reviewed guidance on optimal bowel preparation strategies for these patients. This review summarizes the current data on bowel preparation in patients with surgically altered anatomy, identifies key challenges, addresses existing gaps in knowledge, and presents a tailored approach to bowel preparation in this population based on institutional protocol. In addition, we propose future directions for research and quality improvement to enhance outcomes in this cohort.

## Introduction

Colorectal cancer has been increasing in incidence overall and has become increasingly prevalent among younger adults.^1^^,^^2^ Colonoscopy is the reference standard in colorectal cancer screening and remains an important diagnostic and therapeutic tool in many diseases involving the colon and rectum.^3^ Colonoscopy is often performed in patients with surgically altered anatomy for cancer, inflammatory bowel disease, and diverticular disease.^4^, ^5^, ^6^, ^7^

The number of patients undergoing surveillance after colorectal cancer (CRC) resection has grown, driven by the increasing prevalence of CRC, advancements in surgical techniques and systemic therapies, and an associated improvement in postoperative survival rates.^5^^,^^6^ Colonoscopy after CRC surgery has been linked to reduced overall mortality and a decreased risk of metachronous cancer during the perioperative period.^7^ Accordingly, the U.S. Multi-Society Task Force on CRC and the National Comprehensive Cancer Network recommend a surveillance colonoscopy within the first year after CRC resection.^7^^,^^8^ Ensuring a high-quality colonoscopy is essential for effective surveillance. This includes a complete examination, adequate bowel preparation, and adherence to established quality benchmarks.^9^

Although numerous studies have explored the optimal timing between surgery and surveillance colonoscopy, relatively few have examined the role and quality of bowel preparation in the postresection setting. This review explores the available evidence on bowel preparation in patients with surgically altered anatomy, highlighting major challenges and current knowledge gaps. It outlines a customized bowel preparation strategy informed by our institutional practices and offers recommendations for future research and quality improvement initiatives aimed at optimizing outcomes in this distinct patient population.

## Literature search strategy and study selection

This narrative review was informed by a focused literature search of the PubMed/MEDLINE, Embase, and Web of Science databases from inception through January 2025. The search terms included combinations of “colonoscopy,” “bowel preparation,” “bowel prep,” “colorectal surgery,” “colectomy,” “ileostomy,” “colostomy,” “ileal pouch–anal anastomosis,” and “surgically altered anatomy.”

We included human studies evaluating bowel preparation quality, tolerability, or clinical outcomes in adult patients with prior colorectal surgery, including randomized controlled trials, observational studies, and cohort studies. Pediatric studies, non-English publications, and studies not addressing bowel preparation were excluded. Reference lists of included articles were manually reviewed to identify additional relevant studies. The study selection process is summarized in a Preferred Reporting Items for Systematic reviews and Meta-Analyses flow diagram (Fig. 1).

**Figure 1:**
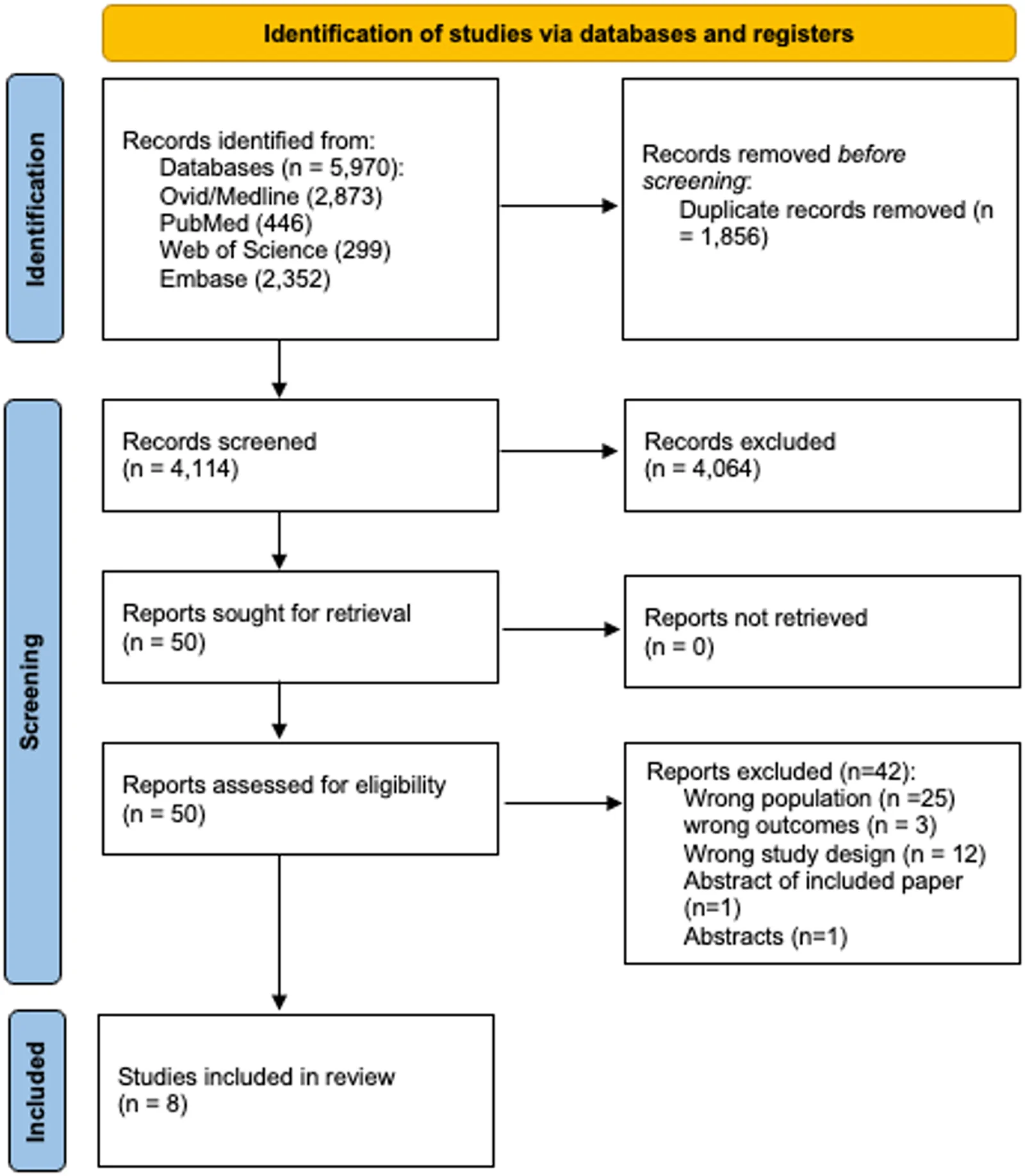
Preferred Reporting Items for Systematic reviews and Meta-Analyses flow diagram illustrating literature search and study selection for bowel preparation in patients with surgically altered bowel anatomy.

## Data on bowel preparation in patients with surgically altered bowel anatomy

### Traditional bowel preparation regimens

Conventional bowel preparation for colonoscopy includes high-volume (≥4 L), low-volume (2-4 L), and ultra-low-volume (<2 L) formulations, which can be administered as split-dose (SD), same-day, or day-before regimens.^10^ According to the U.S. Multi-Society Task Force on CRC, no single purgative has been shown to be superior for average-risk, ambulatory patients. However, 2-L regimens are generally preferred over 4-L preparations due to better tolerability while maintaining comparable cleansing efficacy.^10^

U.S. Food and Drug Administration–approved regimens^10^, ^11^, ^12^ include the following:

(1) 4 L of polyethylene glycol (PEG)-electrolyte solutions (ELSs) (GoLYTELY, CoLyte, GaviLyte).
(2) Sulfate-free 4 L of PEG ELS (NuLYTELY, TriLyte).
(3) Low-volume PEG + ascorbate formulations (2 L of MoviPrep; 1 L of Plenvu [Norgine B.V., Amsterdam, Netherlands]).
(4) Oral sulfate solutions (SUPREP in liquid form or SUTAB tablets).
(5) Sodium picosulfate/magnesium oxide/citric acid (CLENPIQ [Ferring, Parsippany, NJ, USA], formerly Prepopik).
(6) Sodium phosphate tablets (OsmoPrep).

Non–U.S. Food and Drug Administration–approved regimens commonly used in practice include PEG-3350 mixed with a sports drink (MiraLAX [Bayer Healthcare LLC, Hanover, NJ, USA]/Gatorade [Chicago, Ill, USA]), magnesium citrate (two 12-oz bottles plus 2 L of water), and bisacodyl (4 tablets plus 2-3 L of water).^10^ In a large prospective study of 4339 patients, low-volume regimens such as SUPREP, MoviPrep, and MiraLAX/Gatorade achieved significantly higher Boston Bowel Preparation Scale (BBPS) scores than GoLYTELY, while also demonstrating superior tolerability.^13^

Administration and timing are critical for optimal results. The task force strongly recommends SD administration for all patients, with the second portion taken 4 to 6 hours before the procedure and completed at least 2 hours prior.^10^ For example, a randomized trial of 2314 patients comparing 4-L SD PEG versus 2-L SD PEG plus bisacodyl found similar adequacy rates (89% vs 90%), with the low-volume group demonstrating higher willingness to repeat the preparation (91.9% vs 66.9%).^14^

Dietary modifications are typically used alongside purgative regimens. Low-residue or liquid meals are recommended for early and mid-day meals the day before colonoscopy when using SD protocols. For average-risk ambulatory patients, the task force suggests limiting dietary restrictions to the day before the procedure,^10^ although many centers continue to use a full clear-liquid diet the day before. Patient education is also critical, with strategies such as written and verbal instructions, visual aids, booklets, and mobile applications shown to improve bowel preparation quality.^10^

For patients at high risk for inadequate preparation, or those with prior inadequate cleansing, the task force recommends a SD 4 L of PEG ELS plus 15 mg of bisacodyl the afternoon before colonoscopy, combined with a low-residue diet 2 to 3 days beforehand, transitioning to clear liquids the day prior. Additional modifications may include promotility agents, treatment of constipation, and temporary discontinuation of medications that contribute to impaired bowel emptying.^10^

### Bowel preparation scoring systems in postsurgical patients

Several scoring systems are commonly used to assess bowel preparation quality in the general population, including the BBPS, the Aronchick Scale, and the Ottawa Bowel Preparation Scale (OBPS). In patients with surgically altered bowel anatomy, these traditional scales often require modification.

#### BBPS

##### Standard use

The BBPS evaluates bowel cleanliness after optimal suctioning and cleansing in 3 colonic segments: right side of the colon (cecum and ascending colon), transverse colon (including hepatic and splenic flexures), and left side of the colon (descending colon, sigmoid colon, and rectum). Each segment is scored from 0 to 3, and the total score is the sum of all segments.^15^^,^^16^

##### Postsurgical modification

In patients with prior colorectal surgery, only the remaining colonic segments are assessed. For example, after right-sided hemicolectomy, the ascending colon and part of the transverse colon are removed, so the modified BBPS (mBBPS) evaluates the transverse, descending, and rectosigmoid colon. After left-sided hemicolectomy, assessment is limited to the remaining sigmoid colon and rectum. This ensures that the score reflects only the residual colon.^17^^,^^18^ Yoo et al^19^ adopted a different modified version of the BBPS that evaluates the 3 main regions of the remaining colon: proximal, mid, and distal. For instance, in a patient who underwent a right-sided colectomy, bowel cleansing was assessed in the residual colon, divided into the transverse, descending, and rectosigmoid segments.

#### Aronchick Scale

##### Standard use

The Aronchick Scale provides a global assessment of bowel preparation before suctioning, based on residual stool and visible mucosa.

Categories include excellent (>95% mucosa seen), good (>90% mucosa seen), fair (>90% mucosa seen but requiring suctioning), inadequate (<90% mucosa seen but examination completed), and poor (large amount of fecal residue precluding complete examination). Bowel preparation is generally considered satisfactory for “excellent” or “good” grades, and unsatisfactory for “fair” or “poor” grades.^16^

##### Postsurgical modification

Some studies use a simplified classification based on the extent of resection, grouping scores into 3 levels (eg, A1-A2 = excellent/good, A3 = fair, A4-A5 = inadequate/poor) to account for the reduced colonic length and segments.^20^ In other studies, the traditional classification is retained but only applied to the residual colon segments.^21^^,^^22^ This approach is particularly used when segmental scoring is not feasible due to resection. The use of this scale was favored in these studies because the extent of surgical resection precluded the use of segmental scoring.^23^

#### Ottawa Bowel Preparation Scale

##### Standard use

The OBPS evaluates 3 colonic regions (right, mid, and rectosigmoid), each scored 0 to 4, with an additional fluid score of 0 to 2. Higher scores indicate poorer preparation.^23^

##### Postsurgical modification

In patients with prior resections, only the remaining colonic regions are scored.^24^ For instance, after an extended right-sided colectomy, the ascending colon and part of the transverse colon are excluded, and only the mid and left segments are assessed. The total score is adjusted to reflect the remnant colon, with a cutoff (eg, ≤4) defining adequate preparation.

Overall, the use of modified scoring systems in postsurgical patients allows for standardized assessment of bowel preparation quality, which is critical for detecting adenomatous or neoplastic lesions and guiding appropriate oncologic follow-up.^7^^,^^25^ The total scores must be adjusted based on the type and extent of surgical resection, ensuring that the assessment reflects only the residual colon.

### Efficacy of bowel preparation regimens in patients with surgically altered colorectal anatomy

There are a few studies on bowel preparation for colonoscopy in patients with surgically altered bowel anatomy^17^, ^18^, ^19^, ^20^, ^21^, ^22^^,^^24^^,^^26^ (Table 1). In a trial involving patients with previous colorectal surgery, 187 individuals were randomized to receive either a split dose of 4 L of PEG (2 L the evening prior and 2 L the morning of the procedure) or 2 L of PEG on the morning of the procedure alone. The split-dose 4-L regimen was superior, with 85.1% of patients achieving a BBPS score of ≥2 in each segment, compared with 52.7% in the 2-L group.^18^ In another trial involving 120 patients with previous colorectal resection for cancer, 2 different split-dose regimens were compared. Patients were randomized to a low-volume regimen (15 mg of bisacodyl and 2 L of PEG) or a high-volume regimen (4 L of PEG). The difference in bowel preparation adequacy between the groups was negligible, as measured by a modified OBPS score ≤4 (85.0% vs 81.7%, *P* = .624). In this study, a history of left-sided colectomy and a longer time interval since surgery were predictors of inadequate bowel preparation (IBP).^24^

**Table 1:** Summary of published studies on quality of bowel preparation in patients with surgically altered colorectal anatomy

Author, year	Study design	Study location/date	Surgical population	Bowel preparation regimen/comparator groups	Assessment of bowel cleansing efficacy	Number of patients	Primary outcome measures	Key finding	Other findings
Lim, 2012^21^	Single-center, prospective	Korea/October 2009-December 2009	Gastric or colonic resection	4 L of PEG	Aronchick Scale	184 (bowel resection group, n = 92, and a control group, n = 92)	Quality of bowel preparation	Prior bowel resection was associated with significantly worse bowel preparation compared with controls	• Prior resection and obesity were independent predictors of poor preparation • Obese patients with resection had the highest rates of inadequate preparation
Pontone, 2014^20^	Single-center, retrospective case-control	Italy/April 2010-December 2011	Prior bowel resection	SD 4 L of PEG	Aronchick Scale	351 (161 resection group, 190 control group)	Quality of bowel preparation	Patients with prior colorectal resection had significantly higher rates of unsatisfactory bowel preparation than controls (44% vs 12%, *P* < .001)	No difference in fair preparation rates between groups
Mussetto, 2015^24^	Single-center, randomized controlled trial	Italy/July 2013-June 2014	Prior colorectal resection for cancer	Low-volume mixed preparation (15 mg of bisacodyl plus 2 L of PEG solution) vs a standard high-volume preparation (4 L of PEG)	Modified Ottawa Bowel Preparation Scale	120 (60 SD low volume, 60 for high volume)	Quality of bowel preparation	Adequate cleansing rates were similar between low- and high-volume preparations (85.0% vs 81.7%, *P* = .624)	• Low-volume preparation improved right-sided colon cleansing and tolerability • Prior left-sided colectomy and longer time since surgery predicted poor cleansing
Chung, 2017^22^	Single-center, retrospective	Korea/March 2012-April 2014	Colorectal resection	4 L of PEG-containing lavage solution (n = 233) or 2 L of PEG (n = 14) the night before the procedure	Aronchick Scale	247	Adequate bowel preparation rate	Right-sided colon preservation was independently associated with poor bowel preparation on both univariate (22.3% vs 7.5%, *P* = .028) and multivariate analysis (odds ratio: 3.6, 95% confidence interval: 1.0-12.3; *P* = .038)	No association with age, sex, BMI, time since surgery, length of resected colon, comorbidities, or preparation regimen
Liu, 2018^18^	Single-center, randomized controlled trial	China/May 2016-November 2016	Asian patients with prior colorectal surgery for colorectal cancer	Morning-only 2 L of PEG group vs SD 4 L of PEG	Aronchick Scale or modified BBPS	187 (93 in the group for morning-only low-volume regimen [2-L PEG], 94 in the group for SD high-volume regimen [4-L PEG])	Successful bowel preparation rate	The SD high-volume regimen was associated with a significantly higher rate of successful bowel preparation compared with the morning-only low-volume [2-L PEG] regimen (89.4% vs 66.7%, *P* < .001)	• Patient satisfaction was higher with the SD regimen • Polyp and adenoma detection rates were similar between groups
Yoo, 2019^19^	Single-center, retrospective	Korea/March-August 2015	History of colorectal surgery	4 L of PEG or 2 L of PEG with ascorbic acid	Modified BBPS	200 (100 with a history of colorectal surgery vs 100 patients without resection history)	Adequate bowel preparation rate	Adequate bowel cleansing rates were similar between the resection and control groups (modified BBPS 6-9: 88% vs 88%)	• Left-sided colectomy was independently associated with poorer cleansing • Low-volume preparation predicted better bowel cleansing
Lee, 2019^26^	Single-center, retrospective	Korea/June 2013 and June 2016	Colorectal resection due to malignancy	Low-volume combination of 2 bottles of sodium picosulfate with magnesium citrate (10 mg of sodium picosulfate, 3.5 g of magnesium oxide, and 12 g of citric acid), combined with Magcorol (Horii Pharma Industries, Ltd, Osaka, Japan) solution (19.5 g of citric acid and 10.75 g of magnesium carbonate), or a conventional-volume 4-L PEG solution	BBPS	1317 (324 with conventional volume, 993 with low volume)	Adequate bowel preparation rate	Inadequate bowel preparation occurred in 21% of postresection patients, with higher rates among those undergoing early surveillance colonoscopy using low-volume regimens	Colonoscopy within 1 years after surgery and age >80 years independently predicted inadequate preparation
Dioscoridi, 2021^17^	Single-center, retrospective	Italy/January 2016-January 2019	Colorectal resection	N/A	Modified BBPS	1237	Adequate bowel preparation rate	Bowel preparation adequacy varied by type of colorectal resection, with the lowest rates after right-sided colectomy and the highest after total colectomy	Adequate preparation rates ranged from 65.6% to 98.7%, depending on the surgical procedure

*BBPS*, Boston Bowel Preparation Scale; *BMI*, body mass index; *N/A*, not available; *PEG*, polyethylene glycol; *SD*, split dose.

Similarly, a case-control study^19^ involving 200 patients compared low- versus high-volume bowel preparation regimens. In this study, the authors used modified BBPS. There was no statistically significant difference in the adequacy of bowel cleansing between patients with prior colorectal resection and controls (defined as a modified BBPS of 6-9; 88% vs 88%). However, in a logistic regression analysis within the resection group, left-sided colectomy was associated with a significantly reduced likelihood of adequate preparation (odds ratio [OR] = 0.27, *P* = .003), whereas use of a low-volume preparation was independently associated with improved bowel cleansing success (OR = 3.09, *P* = .023). A longer interval between the initial surgery and the colonoscopy was not associated with IBP.^19^

In another study by Chung et al,^22^ bowel preparation quality was assessed in patients with a history of colorectal resection undergoing surveillance colonoscopy. Both univariate and multivariate analyses showed that preservation of the right side of the colon was significantly associated with poorer bowel cleansing quality (22.3% vs 7.5%, *P* = .028; OR, 3.6, 95% confidence interval, 1.0-12.3, *P* = .038).

A retrospective cohort study evaluated 1317 patients who underwent colorectal resection for malignancy and received a surveillance colonoscopy within 1 year postoperatively.^14^ Patients received either a low-volume preparation—a combination of 2 bottles of sodium picosulfate with magnesium citrate (10 mg of sodium picosulfate, 3.5 g of magnesium oxide, and 12 g of citric acid) and Magcorol (Horii Pharma Industries, Ltd, Osaka, Japan) solution (19.5 g of citric acid and 10.75 g of magnesium carbonate)—or a conventional-volume preparation consisting of 4 L of PEG solution. The incidence of IBP was significantly higher in the low-volume group compared with the conventional-volume group (33.1% vs 22.2%, *P* = .03).^26^

In addition, an ongoing clinical trial (NCT02761317) is evaluating the efficacy of bowel preparation in patients with prior colorectal resection by comparing 3 regimens: standard-volume (2-L PEG or 2-L PEG ELS), low-volume (10 mg bisacodyl plus 2-L PEG ELS), and high-volume (4-L PEG ELS).

### Safety and tolerability of bowel preparation in patients with surgically altered colorectal anatomy

Although concerns exist regarding the safety and tolerability of bowel preparation agents in patients with surgically altered colorectal anatomy, available evidence suggests that PEG-based regimens, with or without adjunctive agents such as bisacodyl, are generally safe and well tolerated in this population.

Lim et al^21^ conducted a comparative study of patients with prior colorectal surgery for cancer, evaluating a morning-only low-volume regimen (2-L PEG; n = 93) versus a split-dose high-volume regimen (4-L PEG; n = 94). Overall compliance with bowel preparation instructions was similar between regimens, with no significant difference in poor compliance rates (26.7% vs 30.8%, *P* = .542). Incorrect timing of purgative intake and inadequate dietary restriction occurred at comparable frequencies. Although a higher proportion of patients in the group for the split-dose high-volume regimen (4-L PEG) group consumed less than 80% of the prescribed dose (8.8% vs 1.1%, *P* = .034), most discontinued intake after achieving clear stool effluent rather than due to intolerance, suggesting perceived adequacy of cleansing rather than true nonadherence. Adverse effects—including nausea, vomiting, abdominal discomfort, and distension—were common but did not differ significantly between groups (40.0% vs 34.1%, *P* = .408) and were predominantly mild, with no serious adverse events reported. Despite similar tolerability profiles, patient satisfaction was significantly higher with the split-dose 4-L PEG regimen (93.4% vs 82.2%, *P* = .021), while willingness to repeat the preparation and overall acceptability remained high in both groups.

Similarly, Mussetto et al^24^ conducted a single-center randomized trial in patients with prior colorectal resection for cancer, comparing a low-volume split-dose regimen (15 mg of bisacodyl plus 2 L of PEG; n = 60) with a standard high-volume preparation (4 L PEG; n = 60). The type and severity of preparation-related symptoms were comparable between groups, with no significant differences in tolerability, impact on daily activities, or sleep disturbance. Patients receiving the low-volume regimen reported significantly greater ease of intake (*P* < .001). No preparation-related adverse events occurred in the high-volume group; 1 patient in the low-volume group developed a transient allergic rash after bisacodyl administration, which resolved with oral steroids without affecting completion of the preparation or colonoscopy.

Taken together, these studies indicate that PEG-based bowel preparation regimens—including split-dose and low-volume strategies with adjunctive stimulant laxatives—are generally safe in patients with prior colorectal surgery. Although minor gastrointestinal adverse effects are frequent, they are typically mild, do not differ meaningfully between preparation strategies, and rarely limit regimen completion. Importantly, no serious adverse events have been reported in this population. From a practical standpoint, tolerability may be optimized through split-dose administration, clear patient education regarding expected stool output, adequate hydration, and individualized selection of adjunctive agents. Caution may be warranted with stimulant laxatives in patients with prior hypersensitivity or intolerance; however, available data suggest that such events are rare and self-limited. Overall, existing evidence supports the safety and acceptability of PEG-based bowel preparation regimens in patients with surgically altered colorectal anatomy when appropriately selected and monitored.

### Factors contributing to difficult bowel preparation after gastrointestinal surgery

Bowel preparation is particularly challenging in patients with surgically altered anatomy due to a combination of factors (Fig. 2). These challenges are well documented in the literature and arise from changes in gastrointestinal transit, anatomical alterations that disrupt standard preparation protocols, and gaps in both provider and patient education.

**Figure 2:**
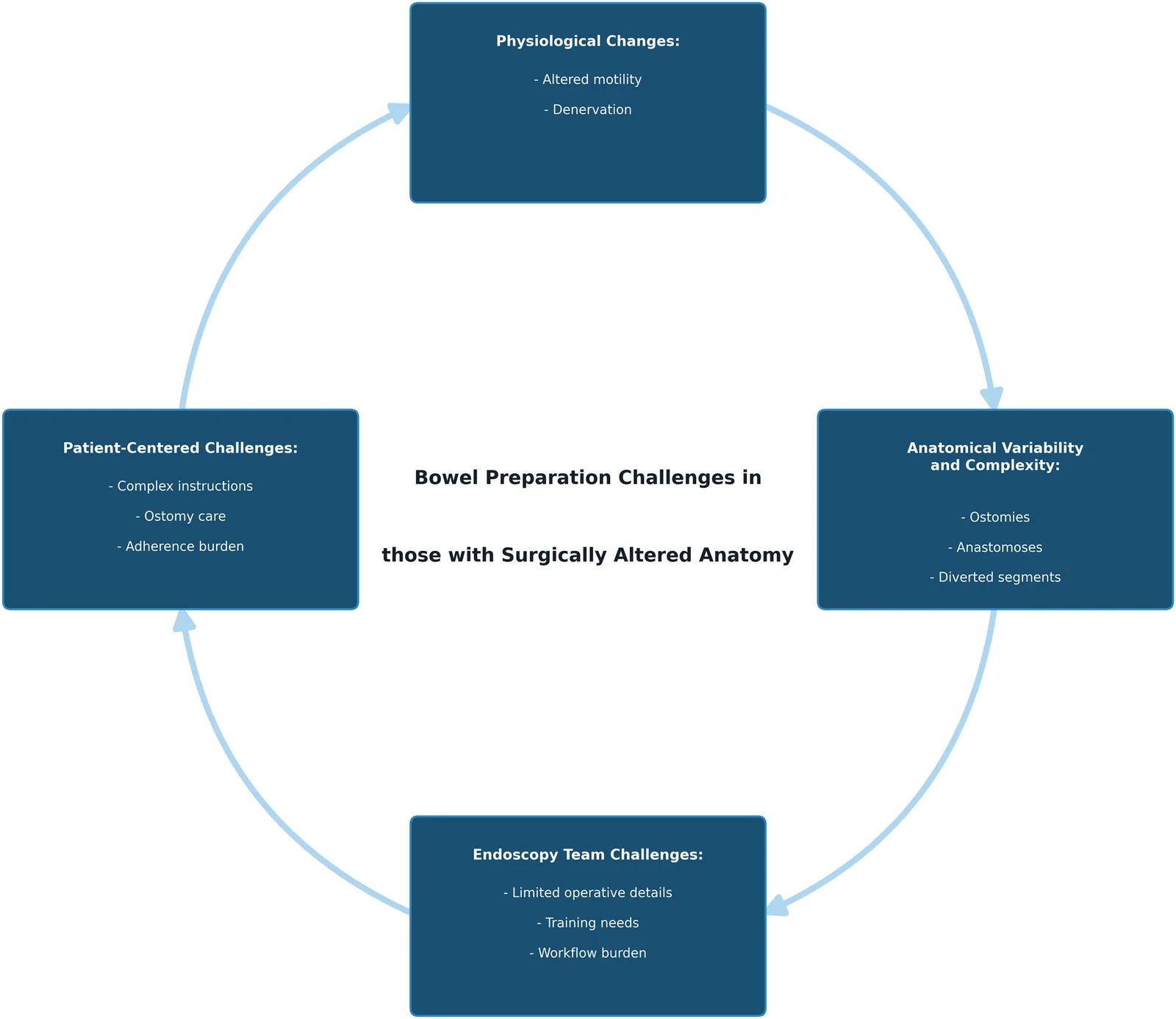
Factors contributing to challenging bowel preparation in patients with surgically altered bowel.

### Physiological changes

Bowel resection and pelvic denervation can significantly impact colonic motility, often resulting in low anterior resection syndrome or anterior resection syndrome. These syndromes are characterized by symptoms such as fecal incontinence, urgency, increased stool frequency, and constipation. More than 90% of patients report changes in bowel function after low anterior resection (LAR),^27^ and approximately 47% experience evacuatory dysfunction.^28^ Severe constipation may result from denervation of the lateral rectal ligaments.^29^ Animal studies support this mechanism; for example, presacral plexus denervation in dogs has been shown to cause decreased colonic motility lasting up to 6 months postoperatively.^30^

This postoperative motility impairment is thought to contribute to the high rates of IBP observed after colorectal surgery.^26^^,^^31^ In a guinea pig model, restoration of bowel motility after bowel anastomosis took approximately 57 to 60 days, as demonstrated by the recovery of phase III migrating motor complex activity and reappearance of key neuropeptides such as vasoactive intestinal peptide, gastrin-releasing peptide, and somatostatin in nerve fibers.^30^ In human studies, the highest incidence of IBP occurs within the first year after surgery, with a subsequent decline attributed to bowel adaptation and gradual improvement in low anterior resection syndrome and anterior resection syndrome symptoms over a 1- to 2-year period.^26^^,^^32^

### Anatomical variability and complexity

After colorectal surgery, patients often have altered anatomy, such as ostomies (ileostomy, colostomy), anastomoses, or diverted bowel segments. These changes can disrupt the normal passage and retention of bowel preparation agents, making it difficult to achieve adequate cleansing. For example, in patients with a diverted colon, stool may remain for extended periods, complicating efforts to clear the bowel before endoscopy. Anatomical variations, such as strictures, angulated anastomoses, or pouches, further complicate the process and may require customized preparation regimens.

Patients with postsurgical anatomy may have complex anatomical configurations—such as diverted segments, anastomoses, or excluded limbs—that are not easily accessible through standard oral preparations. In cases such as Hartmann’s procedure or ileorectal anastomosis, the distal colon or rectum may not be reached by oral purgatives, requiring enema or stoma-directed preparations. The lack of tailored protocols for different surgical reconstructions adds further complexity.

### Increased risk of inadequate preparation

A history of colorectal surgery is recognized as a risk factor for suboptimal bowel preparation.^21^^,^^24^^,^^33^ Logistic regression analysis revealed that prior colorectal surgery significantly increased the risk of IBP, with an OR of 5.170.^34^ IBP is associated with several adverse outcomes, including prolonged procedure duration, decreased adenoma detection rates, reduced cecal intubation success, shortened surveillance intervals, and a 12% to 22% increase in the overall cost of colonoscopy.^35^, ^36^, ^37^, ^38^

Among surgical patients, those with a history of colectomy—particularly segmental resections—are often the most difficult to prepare adequately.^20^^,^^21^ Right-sided colectomy may impair absorption and limit rapid transit, whereas left-sided colectomy may disrupt peristalsis, reducing the effectiveness of luminal content evacuation.^39^ Colorectal surgery may disrupt the enteric nervous system and autonomic innervation, altering normal colonic motility. Changes in both antegrade and retrograde motor patterns have been proposed as potential mechanisms behind impaired bowel cleansing in these patients.^40^^,^^41^

Predictors of IBP in patients with surgically altered bowel anatomy have been rarely investigated (Table 2). Age is a well-recognized factor associated with an increased risk of IBP,^42^^,^^43^ likely due to slower colonic transit and a higher prevalence of obstipation among older adults.^43^^,^^44^ This association was further supported by a multivariate analysis of 1317 patients who underwent colorectal resection for malignancy and subsequent surveillance colonoscopy, in which being over 80 years old emerged as an independent risk factor for IBP.^26^

**Table 2:** Risk factors for inadequate bowel preparation after colorectal surgery

Risk factor	Supporting studies	Proposed mechanism	Practice-based recommendation
Prior colorectal resection	Lim, 2011^21^; Pontone, 2014^20^	Altered anatomy, impaired colonic motility	Use split-dose; escalate if prior IBP
Left-sided colectomy	Mussetto, 2015^24^; Yoo, 2019^19^	Reduced propulsive motility, stool retention	Prefer high-volume or enhanced regimen
Obesity (BMI ≥25 kg/m^2^)	Lim, 2011^21^	Slower colonic transit, higher stool burden	High-volume split-dose preparation
Early surveillance (<1 year postoperatively)	Lee, 2019^26^	Incomplete postoperative bowel adaptation	Avoid low-volume regimens
Advanced age (>80 years)	Lee, 2019^26^	Decreased motility, reduced preparation tolerance	Enhanced instructions ± high volume
Low-volume preparation	Lee, 2019^26^	Insufficient lavage in high-risk anatomy	High-volume split-dose preparation

*BMI*, Body mass index; *IBP*, inadequate bowel preparation.

Specific surgical factors—including left-sided colectomy and preservation of the right side of the colon—have been repeatedly associated with suboptimal preparation, likely reflecting disrupted colonic motility, altered transit patterns, and fecal stasis within remnant segments.^19^^,^^24^

Patient-related factors further compound this risk. Advanced age (>80 years)^26^ and obesity^21^ have been identified as independent predictors of inadequate preparation, potentially due to slower gastrointestinal transit, increased stool burden, and reduced tolerance of bowel preparation regimens. Notably, obese patients with prior bowel resection exhibited particularly high rates of unsatisfactory (79.3%) and poor (37.9%) bowel preparation.^21^ Timing of surveillance colonoscopy also plays a role. Lee et al^26^ reported that colonoscopy performed within the first postoperative year was associated with higher rates of inadequate cleansing, suggesting incomplete physiologic adaptation of the bowel during early recovery. However, other studies reported that longer time since surgery^24^ or the time interval between surgery and colonoscopy^19^^,^^22^ did not consistently influence preparation adequacy, highlighting variability across populations and surgical types.

Preparation-related factors are similarly influential. Low-volume regimens improve tolerability, but multiple studies demonstrate higher rates of inadequate cleansing in high-risk surgical populations, particularly in the early postoperative period. For example, Mussetto et al^24^ compared low-volume mixed preparation (15 mg of bisacodyl plus 2 L of PEG) versus standard high-volume preparation (4 L of PEG) in 120 patients (60 per group). Adequate cleansing rates were similar between low- and high-volume preparations (85.0% vs 81.7%, *P* = .624), although low-volume preparation achieved better right side of the colon cleansing (*P* = .025) and higher tolerability and completion rates (*P* < .001). Another randomized controlled trial comparing a morning-only 2 L of PEG regimen versus a split-dose 4 L of PEG regimen found that the split-dose high-volume regimen (4-L PEG) was associated with a significantly higher rate of successful bowel preparation (89.4% vs 66.7%, *P* < .001).^18^

Surgical approach and type of resection also influence outcomes. In a single-center retrospective study of 1237 patients,^17^ adequate bowel preparation (minimum segmental score ≥2) was achieved in 76.5% of patients after left-sided hemicolectomy, 65.6% after right-sided hemicolectomy, 78.9% after anterior rectal resection, 82.3% after abdominoperineal resection, and 98.7% after total colectomy.

Chemotherapy can impair gastrointestinal motility by damaging enteric nerve endings, thereby altering motor function.^45^ Constipation is the third-most common gastrointestinal symptom among patients receiving chemotherapy, with a reported prevalence of 16%—5% classified as severe and 11% as moderate.^45^^,^^46^ Lee et al^26^ also demonstrated an association between systemic therapy and higher rates of IBP among patients undergoing colonoscopy after colorectal resection.

Although male gender has been associated with a 1.4- to 1.6-fold higher likelihood of IBP in general populations,^47^^,^^48^ this trend was not observed in patients undergoing surveillance colonoscopy after colorectal surgery. The author attributed this to better adherence and increased health consciousness in this surgical cohort, who received regular postoperative follow-up care.^26^

Together, these findings underscore that IBP in postsurgical patients is multifactorial, arising from the interaction among altered anatomy, impaired motility, patient comorbidities, and preparation strategy. These data highlight the need for individualized, anatomy-based bowel preparation protocols tailored to surgical type, patient characteristics, and timing of surveillance colonoscopy.

### Patient-centered challenges

The process of bowel preparation is frequently burdensome and confusing for patients, particularly when instructions are not tailored to their specific surgical history. For example, patients with a diverted colon or rectum may need to administer enemas or irrigate their stoma—tasks that require careful instruction and can be physically and emotionally taxing. The lack of clear, accessible educational materials further compounds these challenges, leaving patients and caregivers uncertain about the correct steps to take. This can result in poor adherence to preparation protocols and increased anxiety about the procedure.

In our experience, providing intelligible, easily accessible educational material to patients and the health care team is an effective intervention to improve the quality of bowel preparation. This is consistent with a meta-analysis,^49^ showing that providing supplementary education to outpatients led to a higher rate of adequate bowel cleansing (79.9% vs 72.9%, *P* < .00001). It also improved preparation quality scores, as reflected by an increase in the BBPS and a decrease in the OBPS. In addition, these interventions reduced colonoscopy withdrawal time and enhanced patient willingness to repeat the procedure (91.9% vs 81.4%, *P* = .004). These findings highlight the value of enhanced patient education in improving both the effectiveness of colonoscopy preparation and overall patient experience.^49^

### Staff perspectives: addressing complex bowel preparation needs

Gastroenterologists and endoscopy nurses face unique challenges when preparing patients with surgically altered anatomy for colonoscopy. One of the primary obstacles is the lack of detailed surgical history or operative reports, which are essential for understanding the patient’s current anatomy and tailoring the bowel preparation accordingly. Without this information, staff may be unaware of critical details such as the type of anastomosis, the presence of strictures, or the configuration of an ostomy.

The variability in surgical techniques and ostomy configurations further complicates the development of a standardized approach to bowel preparation. Each patient may require a customized regimen based on their specific anatomy, which can be time-consuming and complex to implement. For example, patients with a diverted colon or rectum may need bowel preparation administered via the stoma or distal limb, a procedure that requires additional training and resources for the endoscopy team. In addition, the process of educating patients and caregivers about these specialized techniques is often challenging. Staff must be able to clearly communicate complex instructions and provide ongoing support throughout the preparation process. The burden of ensuring adequate bowel cleansing is not only logistical but also emotional, as staff must address patient anxiety and manage expectations about the procedure.

Providing the health care team with easily accessible, standardized educational materials can help mitigate these challenges. When staff are equipped with clear protocols and visual aids, they are better able to guide patients through the preparation process and address any questions or concerns that arise. This approach not only improves the quality of bowel preparation but also enhances the efficiency and confidence of the endoscopy team.

### Bowel preparation considerations based on postsurgical anatomy: our institutional recommendations

Patients with previous colonic resections are frequently excluded from clinical trials, resulting in limited data on the effectiveness and optimal strategies for bowel preparation in this population. Consequently, evidence-based guidelines tailored to this group are lacking. In this review, we outline our approach to tailor colonoscopy bowel preparation based on the type of postsurgical anatomy. In addition, we provide a summarized reference table as educational material for clinicians, patients, and caregivers. Table 3 outlines the various postsurgical anatomical configurations and our corresponding recommendations for bowel preparation. Of note, the following recommendations are derived from institutional protocols and expert consensus, informed by available literature and clinical experience, rather than from high-quality comparative evidence. Formal institutional validation data—such as standardized bowel preparation quality scores, patient tolerability metrics, or adverse event rates—were not systematically collected; therefore, these recommendations represent practice-based guidance rather than outcome-validated protocols.

**Table 3:** Various postsurgical anatomical configurations and our corresponding recommendations for bowel preparation

Type of surgery	Illustration	Diet before procedure	How to complete bowel preparation
Normal colonic anatomy		Clear liquids (water, broths, plain gelatin) Nothing by mouth starting at 12 AM (midnight) procedure day	• Split preparation: Total of 4 L of preparation; total of 2 L starting around 5 PM and 2 L around 5 AM; drink ALL of the preparation.
Abdominal perineal resection			
Low anterior resection after colostomy takedown			
Low anterior resection with diverting loop ileostomy		Regular diet; nothing by mouth starting at 12 AM (midnight) procedure day	Nursing to perform preparation: total of 4 L of preparation brought to the preparation suite the day of your procedure. The nurse will preparation you in the preparation suite before the procedure through intubation of the distal limb of the ostomy with a Foley catheter
Diverting loop ileostomy			
Low anterior resection before colostomy takedown		Clear liquids: water, broths, plain gelatin; nothing by mouth starting at 12 AM (midnight) procedure day	Increased amount of preparation: Total of 5 L of preparation • 4 L of preparation 2 L starting around 5 PM and 2 L around 5 AM, drink ALL of the preparation ■ An additional 1 L will be given the day of procedure by the nurse in the preoperative area through intubation of the distal limb of the ostomy with a Foley catheter
Diverting sigmoid loop colostomy			
End colostomy with Hartmann’s pouch		Clear liquids: water, broths, plain gelatin; nothing by mouth starting at 12 AM (midnight) procedure day	Split preparation: Total of 4 L of preparation • 2 L starting around 5 PM and 2 L around 5 AM, drink ALL of the preparation ■ 2 enemas will be given to you the day of the procedure by the nurse
End ileostomy		Clear liquids: water, broths, plain gelatin; nothing by mouth starting at 12 AM (midnight) procedure day	• Reduced amount of preparation: Total of 1 L of preparation to be taken 4 hours before arriving for your procedure
Ileal pouch-anal anastomosis			

Note: these recommendations are derived from institutional protocols and expert consensus, informed by available literature and clinical experience, rather than from high-quality comparative evidence.

Icons: •Circle denoting patient administered orally at home; ■Square denoting nurse administered in the endoscopy suite.

### Abdominal perineal resection

#### Surgical overview

Abdominal perineal resection (APR) is predominantly used to treat low-lying rectal carcinoma. Other indications include fecal incontinence not amenable to sphincter-sparing procedures or perianal sepsis.^50^ This procedure removes the sigmoid colon, rectum, and anal sphincter and requires the construction of a permanent end colostomy.^51^ Surveillance colonoscopies in patients who have received an APR for rectal cancer are necessary and recommended by numerous institutional societies.^2^

#### Postsurgical impact on bowel preparation

Because the distal colon and rectum are removed, the remaining colon is accessible only via the stoma. Stool consistency is often softer due to shortened transit time. Dehydration risk is low compared with ileostomy patients, but the stoma requires careful handling during preparation.

#### Our institutional bowel preparation recommendation

In patients with an APR, we recommend the standard bowel preparation, including a clear-liquid diet 24 hours before the procedure, nothing by mouth after midnight, and 4 L of PEG given in split dose (2 L the evening before the procedure and 2 L given the day of the procedure).

### Low anterior resection

#### Surgical overview

LAR is performed in patients with locally invasive rectal adenocarcinoma in the middle and upper third of the rectum. This procedure partially or completely removes the rectum but spares the anal sphincter and requires a temporary colostomy for fecal diversion to allow maturation of the anastomosis. The benefit of undergoing an LAR is the sparing of the anal sphincter, which allows patients to undergo a colostomy reversal approximately 3 to 6 months after the initial proctectomy.^52^^,^^53^ Colonoscopy is often performed before ostomy takedown to rule out synchronous lesions and after ostomy takedown for cancer surveillance.

#### Postsurgical impact on bowel preparation

Depending on the presence of a diverting ostomy, the distal colon may not receive oral bowel preparation. Stool may be thinner, and residual secretions can accumulate in the diverted segment. Care must be taken to avoid dehydration when additional preparation is administered.

#### Our institutional bowel preparation recommendation

In patients who are undergoing colonoscopy before colostomy reversal, we recommend that 24 hours before the procedure, patients maintain a clear-liquid diet and nothing by mouth after midnight. A standard 4-L PEG split-dose preparation must be completed by patients at home. Because of altered anatomy, the bowel preparation ingested by mouth will not reach the remaining colon distal to the ostomy. Hence, the patient must bring additional bowel preparation to be administered by the endoscopy nurse before the procedure. We recommend intubation of the distal limb of the ostomy with a Foley catheter and administration of 1 L of PEG in the endoscopy suite to cleanse any inspissated secretions and mucus.

In patients who are undergoing colonoscopy after colostomy reversal, we recommend no changes in the standard bowel preparation protocol.

### Diverting sigmoid loop colostomy

#### Surgical overview

This surgery is indicated when it is necessary to temporarily divert the distal colon or rectum, and restoration of colon continuity is contraindicated or not feasible given the acute clinical condition.^54^ Common indications include emergent decompression of an obstructed or perforated distal colon or rectum, and facilitating the healing of inflammatory conditions distal to the ostomy, such as Crohn’s perianal disease.^55^^,^^56^ Rarely, it is performed for the protection of low-lying colorectal anastomosis with risk of anastomotic failure, a situation in which loop ileostomy is preferred.^57^ In a diverting sigmoid loop colostomy, the ostomy is created by bringing a loop of colon up to the abdominal wall. The proximal limb allows for feces to be excreted, and the distal limb allows mucus and gas to be expelled.^58^

#### Postsurgical impact on bowel preparation

The distal limb is not reached by oral preparation. Stool in the diverted colon is often soft to semiformed. Additional preparation is needed to clear residual mucus and prevent incomplete cleansing.

#### Our institutional bowel preparation recommendation

In these patients undergoing colonoscopy, we recommend the usual clear-liquid diet 24 hours before the procedure and nothing by mouth after midnight. Patients must complete 4 L of PEG split dose at home. Similar to LAR before ostomy takedown, the distal colon needs to be cleansed, and the bowel preparation ingested by mouth will not reach the colon distal to the ostomy. Therefore, 1 additional L of PEG is brought by the patient on the day of the procedure, and it is administered by the nurse in the endoscopy suite.

### End colostomy with Hartmann’s pouch

#### Surgical overview

End colostomy with the Hartmann’s pouch procedure is indicated for patients with pathologies resulting in obstruction or perforation of the left side of the colon, including malignancy, volvulus, trauma, or diverticulitis.^59^^,^^60^ This surgery involves removing the affected part of the distal colon and creating an end colostomy with the proximal end of the excised colon. The distal end is sutured or stapled closed, creating a nonfunctional stump, which is called the Hartmann’s pouch.^61^

#### Postsurgical impact on bowel preparation

The rectal stump may accumulate mucus and secretions, which can obscure visualization. Stool consistency in the proximal colon is often softer due to reduced transit length.

#### Our institutional bowel preparation recommendation

Patients undergoing colonoscopy who have undergone end colostomy with the Hartmann’s pouch should follow a clear-liquid diet 24 hours before the colonoscopy and nothing by mouth after midnight. We recommend a standard 4-L PEG split dose to be taken by the patient at home. The rectal stump is cleaned by a nurse in the endoscopy suite with 2 tap water enemas of 250 mL each.

### Diverting loop ileostomy

#### Surgical overview

Diverting loop ileostomy is performed when it is necessary to temporarily bypass the entire colon and rectum. This is the preferred approach to protect and allow healing of distal colorectal anastomosis.^57^ In patients with inflammatory bowel disease, it is performed to protect ileorectal or ileoanal anastomoses and to divert the flow of intestinal contents in severe, refractory cases.^62^^,^^63^ In this surgery, a loop of the terminal ileum is brought to the anterior abdominal wall and sown to the skin to form a stoma with 2 openings. The proximal limb is connected to the rest of the small bowel and will excrete the intestinal contents; the distal limb remains in continuity with the colon but no longer receives intestinal contents.^64^

#### Postsurgical impact on bowel preparation

The colon receives no oral content; mucus and inspissated secretions can accumulate. Stool consistency is typically liquid or semiliquid in the proximal limb. The risk of dehydration is higher than with colostomy due to high-output ileostomy.

#### Our institutional bowel preparation recommendation

In patients with diverting loop ileostomies who require colonoscopy, patients can maintain a regular diet 24 hours before the procedure and nothing by mouth after midnight. Despite the colon not receiving intestinal contents, it needs to be cleaned of mucous and inspissated secretions. Because of the altered anatomy, bowel preparation will not enter the diverted colon, and enemas are not sufficient to clean the entire colon. Our approach in this scenario is to administer 2 L of PEG in the endoscopy suite 4 hours before the procedure. Two ostomy orifices are identified, and the distal limb is identified, which is typically located more medial and inferior to the proximal limb. In addition, intestinal contents will not exit through the orifice of the distal limb. A Foley catheter is inserted in the distal limb, and the balloon is inflated with 10 mL of sterile water to prevent dislodgement. Bowel preparation is administered at a rate L of 1 to 2 L per hour as tolerated.

### End ileostomy

#### Surgical overview

An end ileostomy is performed when the entire colon, rectum, and anorectal sphincter are removed. The most common indications are ulcerative colitis and familial adenomatous polyposis. These ileostomies are most often permanent, although sometimes can be reversed.^64^^,^^65^ Endoscopic evaluation of the ileum or ileoscopy is usually done to evaluate high ileostomy output, bleeding, and symptoms in patients with inflammatory bowel disease.^66^

#### Postsurgical impact on bowel preparation

The entire colon is absent; only the terminal ileum remains. Stool consistency is liquid. Dehydration risk is high if excessive oral preparation is given.

#### Our institutional bowel preparation recommendation

Patients requiring ileoscopy should maintain a clear-liquid diet 24 hours before the procedure and nothing by mouth after midnight. The amount of bowel preparation can be reduced to 1 L of PEG to be taken at home 4 hours before the procedure.

### Ileal pouch-anal anastomosis

#### Surgical overview

Ileal pouch-anal anastomosis (IPAA), which involves the creation of an ileal pouch, allows the patient to eliminate intestinal contents normally after the removal of the colon and rectum. Patients who undergo this surgery have already undergone total colectomy, most commonly due to ulcerative colitis and familial adenomatous polyposis.^67^ Endoscopic evaluation of the ileoanal pouch, commonly known as pouchoscopy, is performed for surveillance of polyps arising from the rectal cuff and for the assessment of inflammatory bowel disease.^68^^,^^69^

#### Postsurgical impact on bowel preparation

Pouch contents are liquid, and mucus can accumulate in the ileal reservoir. Overly aggressive preparation may lead to dehydration or electrolyte imbalance.

#### Our institutional bowel preparation recommendation

Patients undergoing pouchoscopy should maintain a clear-liquid diet 24 hours before the procedure and nothing by mouth after midnight. We recommend that the amount of bowel preparation be reduced to 1 L of PEG to be taken at home 4 hours before the procedure.

### Special considerations and areas of uncertainty

The agent, dosage, and timing of bowel preparation recommended are based on our experience and expert opinion, given the lack of scientific literature on the topic. PEG is our preferred agent, although newer, lower-volume preparations can be considered. Regimens including bisacodyl to decrease the volume of PEG are also effective, as shown by the Musetto et al trial.^24^ Nonetheless, bisacodyl-based regimens should be avoided in patients with diverting loop ileostomy, end ileostomy, and IPAA. Bisacodyl acts locally in the colon by directly enhancing motility and increasing stool water; the action of bisacodyl on the small intestine is negligible.^70^

We acknowledge that intolerance to PEG is often reported by patients when taken orally; however, these complaints are rare when administered directly into the ostomy. We perform the bowel preparation of the diverted colon in the endoscopy unit on the same day of the procedure. We use a summarized chart with illustrations of each postsurgical anatomy for guidance of our endoscopy team. It is uncertain if this can be performed the day before in clinic or at home in motivated patients and caregivers. Regardless of the approach, education of nurses, patients, and caregivers is paramount to ensure high-quality bowel preparation.

It is worth noting that alternative PEG-based regimens, including low-volume PEG with ascorbate, PEG 3350–based preparations, and tablet-based formulations such as SUTAB, may be considered based on patient tolerance and institutional practice. Adjunctive strategies—including simethicone, dietary modification, and enema- or irrigation-based approaches—may be useful in selected scenarios, particularly when oral preparation does not adequately reach diverted bowel segments. However, our experience with these alternative regimens is limited, and robust prospective data are needed to better define their efficacy and safety in patients with surgically altered anatomy.

### Future directions

Moving forward, there is a clear need for the establishment of multicenter registries and quality improvement initiatives focused on bowel preparation in patients with surgically altered anatomy. Given the heterogeneity of surgical procedures and the variability in institutional practices, collaborative efforts could generate robust data to inform evidence-based protocols. Such registries would enable the identification of best practices, enhance benchmarking efforts, and ultimately lead to improved patient outcomes and procedural efficiency.

In addition to institutional-level changes, the development of standardized patient-facing educational tools is essential. Tailored handouts that account for anatomical alterations, cultural context, and literacy levels—particularly in underserved or linguistically diverse populations—could improve patient understanding and compliance with bowel preparation protocols. Mobile health applications, equipped with interactive instructions, reminders, and troubleshooting support, represent a promising avenue for scalable, personalized patient education. These tools could be co-designed with input from patients and clinicians to maximize usability and impact.

There are also several unanswered research questions that merit future investigation. One critical question is whether bowel preparation in this population can be reliably performed at home, or whether inpatient settings offer significantly better outcomes. This has important implications for resource utilization and patient convenience. In addition, studies comparing different bowel preparation agents, risk factors for IBP, and delivery methods—particularly via ostomy—are needed to identify the most effective and well-tolerated options. As we work to refine protocols, these research avenues will help optimize care for this growing and complex patient population.

### Summary

In conclusion, colonoscopy remains an essential procedure for screening, diagnosis, and therapy of patients after colorectal surgery. Bowel preparation of the diverted colon is vital and should be individualized to the patient postsurgical anatomy. We recommend PEG-based formulas to be taken orally and to clean the diverted colon via ostomy. Bisacodyl-based regimens should not be used in patients with diverting loop ileostomy, end ileostomy, and IPAA. We administer PEG through the ostomy on the same day of the procedure with the help of trained nurses in the endoscopy unit. Education of patients and caregivers is an essential part of the process. We hope this review and educational material are useful to guide clinicians in this common clinical scenario.

## Patient consent

This article does not discuss individual patients, so no consent was needed.

## Disclosure

All authors disclosed no financial relationships.

## References

[1] Scholefield J.H., Steele R.J., British Society For Gastroenterology; Association of Coloproctology for Great Britain and Ireland (2002). Guidelines for follow up after resection of colorectal cancer. Gut.

[2] Kennedy E., Zwaal C., Asmis T. (2022). An evidence-based guideline for surveillance of patients after curative treatment for colon and rectal cancer. Curr Oncol.

[3] Schultz J.K., Azhar N., Binda G.A. (2020). European Society of Coloproctology: guidelines for the management of diverticular disease of the colon. Colorectal Dis.

[4] Clarke W.T., Feuerstein J.D. (2019). Colorectal cancer surveillance in inflammatory bowel disease: practice guidelines and recent developments. World J Gastroenterol.

[5] (2022). GBD 2019 Colorectal Cancer Collaborators. Global, regional, and national burden of colorectal cancer and its risk factors, 1990-2019: a systematic analysis for the Global Burden of Disease Study 2019. Lancet Gastroenterol Hepatol.

[6] Arnold M., Sierra M.S., Laversanne M. (2017). Global patterns and trends in colorectal cancer incidence and mortality. Gut.

[7] Kahi C.J., Boland C.R., Dominitz J.A. (2016). Colonoscopy surveillance after colorectal cancer resection: recommendations of the US Multi-Society Task Force on colorectal cancer. Gastrointest Endosc.

[8] Benson A.B., Venook A.P., Cederquist L. (2017). Colon cancer, version 1.2017, NCCN Clinical Practice Guidelines in Oncology. J Natl Compr Canc Netw.

[9] Rex D.K., Anderson J.C., Butterly L.F. (2024). Quality indicators for colonoscopy. Am J Gastroenterol.

[10] Jacobson B.C., Anderson J.C., Burke C.A. (2025). Optimizing bowel preparation quality for colonoscopy: consensus recommendations by the US Multi-Society Task Force on colorectal cancer. Am J Gastroenterol.

[11] Johnson D.A., Barkun A.N., Cohen L.B., US Multi-Society Task Force on Colorectal Cancer (2014). Optimizing adequacy of bowel cleansing for colonoscopy: recommendations from the US Multi-Society Task Force on colorectal cancer. Gastroenterology.

[12] Hagen R., Nguyen M.T.T., Anderson J.C. (2025). Navigating bowel preparation for colonoscopy: a comprehensive overview. J Clin Gastroenterol.

[13] Gu P., Lew D., Oh S.J. (2019). Comparing the real-world effectiveness of competing colonoscopy preparations: results of a prospective trial. Am J Gastroenterol.

[14] Barkun A.N., Martel M., Epstein I.L. (2022). The bowel CLEANsing national initiative: high-volume split-dose vs low-volume split-dose polyethylene glycol preparations: a randomized controlled trial. Clin Gastroenterol Hepatol.

[15] Lai E.J., Calderwood A.H., Doros G. (2009). The Boston Bowel Preparation Scale: a valid and reliable instrument for colonoscopy-oriented research. Gastrointest Endosc.

[16] Parmar R., Martel M., Rostom A. (2016). Validated scales for colon cleansing: a systematic review. Am J Gastroenterol.

[17] Dioscoridi L., Forti E., Pugliese F. (2021). A modified Boston Bowel Preparation Scale after colorectal surgery. Ann Coloproctol.

[18] Liu Z., Li Y.Y., Luo X.T. (2018). Split-dose 4-L polyethylene glycol regimen for patients with previous colorectal surgery in bowel preparation before colonoscopy: a randomized, controlled, single-blind study. J Dig Dis.

[19] Yoo I.K., Jeen Y.T., Choi S.J. (2019). Evaluation of bowel preparation quality in patients with a history of colorectal resection. Turk J Gastroenterol.

[20] Pontone S., Leonetti G., Lamazza A. (2014). A retrospective case-control study evaluating the bowel preparation quality during surveillance colonoscopy after colonic resection. ISRN Gastroenterol.

[21] Lim S.W., Seo Y.W., Sinn D.H. (2012). Impact of previous gastric or colonic resection on polyethylene glycol bowel preparation for colonoscopy. Surg Endosc.

[22] Chung E., Kang J., Baik S.H. (2017). Impact of resected colon site on quality of bowel preparation in patients who underwent prior colorectal resection. Surg Laparosc Endosc Percutan Tech.

[23] Rostom A., Jolicoeur E. (2004). Validation of a new scale for the assessment of bowel preparation quality. Gastrointest Endosc.

[24] Mussetto A., Frazzoni L., Paggi S. (2015). Split dosing with a low-volume preparation is not inferior to split dosing with a high-volume preparation for bowel cleansing in patients with a history of colorectal resection: a randomized trial. Endoscopy.

[25] le Clercq C.M.C., Winkens B., Bakker C.M. (2015). Metachronous colorectal cancers result from missed lesions and non-compliance with surveillance. Gastrointest Endosc.

[26] Lee D., Chun H.-K. (2019). Bowel preparation for surveillance colonoscopy after colorectal resection: a new perspective. Ann Coloproctol.

[27] Dennett E.R., Parry B.R. (1999). Misconceptions about the colonic J-pouch: what the accumulating data show. Dis Colon Rectum.

[28] Keane C., Wells C., O'Grady G. (2017). Defining low anterior resection syndrome: a systematic review of the literature. Colorectal Dis.

[29] Speakman C.T., Madden M.V., Nicholls R.J. (1991). Lateral ligament division during rectopexy causes constipation but prevents recurrence: results of a prospective randomized study. Br J Surg.

[30] Ishikawa M., Mibu R., Iwamoto T. (1997). Change in colonic motility after extrinsic autonomic denervation in dogs. Dig Dis Sci.

[31] Cooper G.S., Chak A., Koroukian S. (2005). The polyp detection rate of colonoscopy: a national study of Medicare beneficiaries. Am J Med.

[32] Pedersen I.K., Christiansen J., Hint K. (1986). Anorectal function after low anterior resection for carcinoma. Ann Surg.

[33] Hassan C., Fuccio L., Bruno M. (2012). A predictive model identifies patients most likely to have inadequate bowel preparation for colonoscopy. Clin Gastroenterol Hepatol.

[34] Zhang N., Xu M., Chen X. (2024). Establishment of a risk prediction model for bowel preparation failure prior to colonoscopy. BMC Cancer.

[35] Rex D.K., Imperiale T.F., Latinovich D.R. (2002). Impact of bowel preparation on efficiency and cost of colonoscopy. Am J Gastroenterol.

[36] Sharma P., Burke C.A., Johnson D.A. (2020). The importance of colonoscopy bowel preparation for the detection of colorectal lesions and colorectal cancer prevention. Endosc Int Open.

[37] Pantaleón Sánchez M., Gimeno Garcia A.-Z., Bernad Cabredo B. (2022). Prevalence of missed lesions in patients with inadequate bowel preparation through a very early repeat colonoscopy. Dig Endosc.

[38] Guo R., Wang Y.-J., Liu M. (2019). The effect of quality of segmental bowel preparation on adenoma detection rate. BMC Gastroenterol.

[39] Phillips S.F. (1984). Functions of the large bowel: an overview. Scand J Gastroenterol Suppl.

[40] Mañé N., Jimenez M. (2014). Interplay between myogenic pacemakers and enteric neurons determine distinct motor patterns in the rat colon. Neurogastroenterol Motil.

[41] Vather R., O'Grady G., Arkwright J.W. (2016). Restoration of normal colonic motor patterns and meal responses after distal colorectal resection. Br J Surg.

[42] Duncan J.E., Sweeney W.B., Trudel J.L. (2006). Colonoscopy in the elderly: low risk, low yield in asymptomatic patients. Dis Colon Rectum.

[43] McNabb-Baltar J., Dorreen A., Al Dhahab H. (2016). Age is the only predictor of poor bowel preparation in the hospitalized patient. Can J Gastroenterol Hepatol.

[44] Loffeld R.J., Liberov B., Dekkers P.E. (2012). Yearly diagnostic yield of colonoscopy in patients age 80 years or older, with a special interest in colorectal cancer. Geriatr Gerontol Int.

[45] McQuade R.M., Stojanovska V., Abalo R. (2016). Chemotherapy-induced constipation and diarrhea: pathophysiology, current and emerging treatments. Front Pharmacol.

[46] Yamagishi A., Morita T., Miyashita M. (2009). Symptom prevalence and longitudinal follow-up in cancer outpatients receiving chemotherapy. J Pain Symptom Manage.

[47] Lebwohl B., Wang T.C., Neugut A.I. (2010). Socioeconomic and other predictors of colonoscopy preparation quality. Dig Dis Sci.

[48] Chan W.-K., Saravanan A., Manikam J. (2011). Appointment waiting times and education level influence the quality of bowel preparation in adult patients undergoing colonoscopy. BMC Gastroenterol.

[49] Peng S., Liu S., Lei J. (2022). Supplementary education can improve the rate of adequate bowel preparation in outpatients: a systematic review and meta-analysis based on randomized controlled trials. PLoS One.

[50] Menon G., Wei R., Bamford R. (2025). http://www.ncbi.nlm.nih.gov/books/NBK574568.

[51] Perry W.B., Connaughton J.C. (2007). Abdominoperineal resection: how is it done and what are the results?. Clin Colon Rectal Surg.

[52] Kokuba Y., Sato T., Ozawa H. (2006). Laparoscopic low anterior resection for rectal cancer. Surg Technol Int.

[53] Ridolfi T.J., Berger N., Ludwig K.A. (2016). Low anterior resection syndrome: current management and future directions. Clin Colon Rectal Surg.

[54] Lightner A.L., Pemberton J.H. (2017). The role of temporary fecal diversion. Clin Colon Rectal Surg.

[55] Doughty D. (2005). Principles of ostomy management in the oncology patient. J Support Oncol.

[56] Plasencia A., Bahna H. (2019). Diverting ostomy: for whom, when, what, where, and why. Clin Colon Rectal Surg.

[57] Rondelli F., Reboldi P., Rulli A. (2009). Loop ileostomy versus loop colostomy for fecal diversion after colorectal or coloanal anastomosis: a meta-analysis. Int J Colorectal Dis.

[58] Zoccali M.B., Kuritzkes B.A., Hyde G.A. (2020). Laparoscopic fecal diversion (loop sigmoid colostomy). Dis Colon Rectum.

[59] Hallam S., Mothe B.S., Tirumulaju R. (2018). Hartmann's procedure, reversal and rate of stoma-free survival. Ann R Coll Surg Engl.

[60] Barbieux J., Plumereau F., Hamy A. (2016). Current indications for the Hartmann procedure. J Visc Surg.

[61] Choubey R.P., Patnaik P.K., Sharma A.K. (2011). Hartmann's procedure revisited. Med J Armed Forces India.

[62] Shirley F., Kodner I.J., Fry R.D. (1984). Loop ileostomy. Techniques and indications. Dis Colon Rectum.

[63] Hanna M.H., Vinci A., Pigazzi A. (2015). Diverting ileostomy in colorectal surgery: when is it necessary?. Langenbecks Arch Surg.

[64] Rajaretnam N., Lieske B. (2025). http://www.ncbi.nlm.nih.gov/books/NBK519003.

[65] Whitehead A., Cataldo P.A. (2017). Technical considerations in stoma creation. Clin Colon Rectal Surg.

[66] Shen B. (2016). The evaluation of postoperative patients with ulcerative colitis. Gastrointest Endosc Clin N Am.

[67] Ng K.-S., Gonsalves S.J., Sagar P.M. (2019). Ileal-anal pouches: a review of its history, indications, and complications. World J Gastroenterol.

[68] van der Ploeg V.A., Maeda Y., Faiz O.D. (2018). Standardising assessment and documentation of pouchoscopy. Frontline Gastroenterol.

[69] McLaughlin S.D., Clark S.K., Thomas-Gibson S. (2009). Guide to endoscopy of the ileo-anal pouch following restorative proctocolectomy with ileal pouch-anal anastomosis; indications, technique, and management of common findings. Inflamm Bowel Dis.

[70] Corsetti M., Landes S., Lange R. (2021). Bisacodyl: a review of pharmacology and clinical evidence to guide use in clinical practice in patients with constipation. Neurogastroenterol Motil.

